# Editorial: Knock-on mental health effects of substance and drug use as a coping strategy

**DOI:** 10.3389/fpubh.2022.1110781

**Published:** 2022-12-23

**Authors:** Yuka Kotozaki

**Affiliations:** Iwate Medical University, Yahaba, Japan

**Keywords:** drug use, stress, coping strategy, mental health, addiction risk

This Research Topic was designed to explore the *Knock-on mental health effects of substance and drug use as a coping strategy*. Often people will seek to relieve or evade the adverse emotions that are elicited from stressful, overwhelming, and unpredictable life situations (1). A common coping strategy, that is applicable in varying contexts, is substance and drug use (1, 2). However, addictions to these coping methods can develop, and in turn contribute to the development of comorbid mental disorders, including anxiety and depression (3). It is important to emphasize that if substance or drug use can provide short-term relief from negative emotions and situations to individuals as a coping strategy, the long-term effects on an individual's mental health can be harmful and lasting. Six of the papers submitted to the journal by international researchers were deemed suitable for publication after a thorough peer review process. The following is a summary of the main results of each of these manuscripts.

In the first article of this Research Topic, Bonsaksen et al. examined the use of alcohol and addictive drugs during the coronavirus disease 2019 (COVID-19) outbreak in Norway and examine their association with mental health problems and problems related to the pandemic. Daily use of alcohol was associated with depression and expecting financial loss in relation to the COVID-19 outbreak. The use of cannabis was associated with expecting financial loss in relation to COVID-19. The use of sedatives was associated with anxiety, depression, and insomnia. Use of painkillers was associated with insomnia and self-reported risk of complications if contracting the coronavirus. They suggested that the occurrence of mental health problems is more important for an understanding of the use of alcohol and addictive drugs during the COVID-19 outbreak in Norway, compared to specific pandemic-related worries.

In the next article, Mougharbel et al. described the association between socio-demographic, and COVID-19-related worries and combinations of concurrent alcohol use, depression, and anxiety in a sample of Canadian adults during the COVID-19 pandemic. Several demographic and COVID-19-related worries for increased odds of alcohol intake and co-morbid psychological distress during the COVID-19 pandemic, including identifying as a woman, high-income groups, being divorced, separated or widowed, and experiencing financial worries and COVID-19 illness worries. They suggest these characteristics should be considered when developing prevention and treatment programs for adults with problematic alcohol use and comorbid anxiety and depression.

In the third article of this Research Topic, Hassen et al. examined khat use, psychological problems, and motivation to change and to determine associated factors of khat use among students from Jimma University seeking psychological assistance. Subjects showed high khat use in the past month and 17.0% showed highly problematic use. Also, subjects were extremely burdened with comorbid psychiatric problems: 21.6% reported functioning problems due to past mental disorders, 60.2% scored above the cut-off for current common mental disorders, 37.9% screened positive for posttraumatic stress disorder, and 47.1% reported hazardous alcohol use. Additionally, higher motivation to change khat use was associated with higher use of the substance. They suggested the challenges of studying substance use and the need for planning interventional strategies for khat as well as for comorbid mental health problems among university students in the countries of the khat belt.

In the next article, Ahmed et al. examined that stigma, social support, and other determinants of anxiety and depression in people living with HIV/AIDS (PLWHA) in Pakistan. In PLWHA, the prevalence of co-morbid depression and anxiety was 80%. Separately, 89.9% had depression, and 80.3% had anxiety. Use of illicit drugs, low social support, being male, and HIV-related stigma were significant predictors of depression. Having detectable viral load, young age, no formal education, low or moderate social support, addiction to illicit drugs, and HIV stigma had a remarkable association with anxiety. They suggested that given the high prevalence of anxiety and depression among PLWHA, it should focus more on monitoring mental health, expanding mental health services, and developing interventions based on identified factors to treat depression and anxiety among PLWHA.

Wang et al. described single-photon emission computed tomography (SPECT) findings on neuropsychiatric symptoms caused by nitrous oxide (N_2_O) abuse. The clinical manifestations of the 16 patients with neuropsychiatric symptoms were mood lability, anxiety, hallucination, delusion, agitation, confusion, and other psychiatric symptoms. In addition, 15 of the patients also complained of memory decline; 14 patients manifested numbness or paresthesia; 14 patients developed limb weakness, and their motor impairments were more severe in the lower limbs than in the upper limbs; and eight patients had urinary and defecation disturbances. Also, SPECT showed hypoperfusion in the frontal and temporal lobes, which is consistent with the clinical findings. They suggest the obvious effect of N_2_O abuse on cerebral blood flow (CBF) in patients with neuropsychiatric symptoms and CBF perfusion imaging is helpful to detect the changes in the local brain functional activity in patients with N_2_O abuse.

Finally, in a systematic review, Dominik et al. assessed potential and frequently used drugs for Pharmacological Neuroenhancement (PN) and their clinical and especially pro-cognitive effects and side effects and integrated these drugs into legal categories based on the German legal system. They suggested to elucidate the German legal situation, PN substances have to be divided into over-the-counter drugs, prescription drugs, and illegal drugs.

In conclusion, the editor wishes to thank all the authors, the reviewers, and the Editorial board members for contributing to this Research Topic. I hope this Research Topic might inspire future and novel research approaches in the field of mental health.

## Author contributions

The author confirms being the sole contributor of this work and has approved it for publication.
